# Lovastatin Inhibits Low Molecular Weight Hyaluronan Induced Chemokine Expression via LFA-1 and Decreases Bleomycin-Induced Pulmonary Fibrosis

**Published:** 2014-09

**Authors:** Mark J. Hamblin, Michael Eberlein, Katharine Black, Robert Hallowell, Samuel Collins, Yee Chan-Li, Maureen R. Horton

**Affiliations:** 1 University of Kansas Hospital, USA;; 2 University of Iowa, Iowa City, IA, USA;; 3 Massachusetts General Hospital, Boston, MA, USA;; 4 Johns Hopkins University School of Medicine, Baltimore, MD, USA

**Keywords:** Hyaluronan, Lovastatin, LFA-1, Fibrocytes, Bleomycin

## Abstract

**BACKGROUND:**

Lovastatin has a unique ability to bind Leukocyte Function Antigen-1 (LFA-1), an integrin necessary for the full expression of inflammatory cytokines induced by the low molecular weight form of the extracellular matrix glycosaminoglycan hyaluronan (LMW HA). We hypothesized that lovastatin could inhibit LMW HA inflammatory signals via interaction with LFA-1, and attenuate bleomycin induced pulmonary fibrosis.

**METHODS:**

We evaluated the effects of lovastatin, pravastatin, LFA-1 blocking antibodies, and a novel LFA-1 inhibitor LFA 878 on LMW HA induced cytokine production in alveolar macrophages. We evaluated the effect of lovastatin in a bleomycin model of lung injury.

**RESULTS:**

Lovastatin immediately inhibited the LMW HA induced cytokine MIP 1-α (*p*=0.001) independent of HMG CoA reductase. Pravastatin showed no inhibitory profile when administered simultaneously with LMW HA. LFA-1 blocking antibodies and the small molecule statin derivative LFA 878 showed an inhibitory profile similar to lovastatin. Lovastatin showed decreased fibrosis on histopathology and improved survival at day 14, with a decrease in fibrocytes noted at day 8.

**CONCLUSION:**

Lovastatin and LFA 878 inhibit LMW HA inflammatory signaling independent of HMG-CoA decreasing the chemotactic cytokine MIP 1-α. Lovastatin treatment improves survival in bleomycin lung injury with decreased fibrocytes and fibrosis.

## INTRODUCTION

Since their introduction as cholesterol lowering agents, HMG CoA reductase Inhibitors or statins, have been found to have potent anti-inflammatory properties (1, 2). Most of these effects have been attributed to the downstream consequences of inhibiting HMG CoA reductase (3-5). However, the lipophilic statins, such as lovastatin and simvastatin, also exhibit the ability to directly inhibit activity of the leukocyte integrin α_L_β_2_, or leukocyte function antigen-1 (LFA-1) (6). LFA-1 is found on all white blood cells, as the heterodimer of the plasma membrane receptors CD11a and CD18, and it serves as the primary binding site for ICAM-1, allowing for activation and migration of leukocytes on endothelial and tissue surfaces (7). LFA-1 has also been linked to cell signaling properties with various hyaluronan receptors, including the toll-like receptors (TLR2 and TLR4) and CD-44, which have been linked to the pathogenesis of interstitial pulmonary fibrosis (8-10).

Hyaluronan (HA) is a simple glycosaminoglycan with repeating units of D-glucuronic acid and D-N-acetylglucosamine. It is synthesized into a high molecular weight molecule (HMW HA) found in the extracellular matrix of cells throughout the body including the lung where it encompasses almost 70% of the proteoglycan content of the lung parenchyma (11). In its high molecular weight isoform, HA is a relatively inert glycosaminoglycan regulating water homeostasis and optimizing protein trafficking to the cell surface. However, in response to tissue injury, shear stress, or oxygen free radical damage, HMW HA depolymerizes to low molecular weight fragments (LMW HA) capable of activating various cell lines of the innate immune system, as well as enhancing T cell responses by activating and up-regulating co-stimulatory molecules on dendritic cells (12-19) . Markedly elevated levels of HA are found in bronchoalveolar lavage specimens from patients with fibrotic lung injuries ranging from sarcoidosis to idiopathic pulmonary fibrosis (20-22). A similar up-regulation of LMW HA is also found in bleomycin injured lungs (23, 24). The role of LMW HA in the pathogenesis of inflammatory interstitial lung diseases leading to pulmonary fibrosis is still emerging; however, given that LFA-1 has been associated with the HA receptors CD44 and TLR2, we hypothesized that inhibition of LFA-1 could be exploited to treat inflammatory interstitial lung diseases leading to pulmonary fibrosis.

## MATERIALS AND METHODS

### Cells and cell lines

The MH-S murine alveolar macrophage cell line (American Type Culture Collection, Rockville, MD, USA) was maintained per the manufacturer’s guidelines. Thioglycollate-elicited peritoneal macrophages were lavaged from female C57BL/6 mice (The Jackson Laboratory, Bar Harbor, ME, USA), 4 days after injection of 3 ml of sterile thioglycollate (Sigma-Aldrich, St. Louis, MO, USA). All cells were allowed to adhere overnight in DMEM media supplemented with 10% heat-inactivated low-LPS FBS and 1% penicillin-streptomycin/1% glutamine before use. For experiments, cells were washed in phosphate-buffered saline and stimulated in DMEM media with 1% glutamine, 1% penicillin/ streptomycin. To exclude the effects of contaminating LPS on experimental conditions, cell stimulation was conducted in serum free DMEM in the presence of polymixin B 10 ug/ml (Sigma-Aldrich). All animal experiments were approved by the Johns Hopkins Committee on Animal Use (Baltimore, MD, USA), and experiments were conducted in accordance with their guidelines and regulations.

### Antibodies and reagents

Purified low molecular weight hyaluronan fragments from human umbilical cords (Calbiochem Novabiochem, Billerica, MA, USA) are free of protein and other glycosaminoglycans with a peak molecular weight of 200,000 Da. Stock solutions of reagents were tested for LPS contamination using the *Limulus* amebocyte assay (Sigma-Aldrich). Lovastatin and Pravastatin were purchased from Sigma-Aldrich. LFA 878 was a generous gift from Novartis (Basel-Switzerland). Inhibitory antibodies included H300, Y17, M17/4 (Santa Cruz Biotechnology, Santa Cruz, CA, USA. PMA, Ionomycin, Flow cytometry antibodies and reagents were purchased from BD (Franklin Lakes, NJ, USA). Collagen antibody was purchased from Abcam (Cambridge, MA, USA).

### Northern Analysis of mRNA Production

RNA was extracted from confluent cell monolayers using 4 M guanidine isothiocyanate and purified by centrifugation through 5.7 M cesium chloride for 12–18 h at 35,000 rpm as described (25) . Ten μg of total RNA was electrophoresed under denaturing conditions through a 1% formaldehyde-containing agarose gel and RNA was transferred to Nytran (Schleicher and Schuell) hybridization filters. Blots were briefly rinsed in 5× SSC, RNA was cross-linked to the filter by UV cross-linking (Stratagene, La Jolla, CA), and blots were hybridized overnight with 10^6^ cpm/ml of ^32^P-labeled DNA labeled by the random prime method (Amersham Pharmacia Biotech). Following hybridization, blots were washed once in 2× SSC/0.1% SDS at room temperature for 30 min with shaking, and then washed twice in 0.1× SSC/0.1% SDS at 50°C with shaking for 20 min each wash. Blots were exposed at −70°C against Kodak XAR diagnostic film. Differences in RNA loading were documented by hybridizing selected blots with ^32^P-labeled cDNA for aldolase (26) . Densitometric scanning was performed using a Molecular Dynamics Personal Densitometer SI (Sunnyvale, CA).

### ELISA for protein secretion

Cells (either MH-S Alveolar Macrophages or Thioglycollate elicted peritoneal macrophages) were allowed to adhere overnight in DMEM media supplemented with 10% heat-inactivated low-LPS FBS and 1% penicillin-streptomycin/1% glutamine in a 6 or 12 well plate before use. Cells were then washed in phosphate-buffered saline and stimulated in DMEM media with 1% glutamine, 1% penicillin/ streptomycin. To exclude the effects of contaminating LPS on experimental conditions, cell stimulation was conducted in serum free DMEM in the presence of polymixin B 10 ug/ml. Culture media (protein supernatant) was collected and analyzed by ELISA for cytokine protein production following an 18-hour stimulation determined by prior experiments indicating peak protein production. ELISAs for MIP-1α, KC, TNF-α, and RANTES were performed according to the manufacturer’s guidelines (R&D Systems, Minneapolis, MN, USA). Colorimetric changes were measured in an ELISA plate reader and analyzed with Microplate Manager III (Bio-Rad) software.

### Animal Experiments

18-20 week old female C57BL/6 background mice were used for all animal experiments. For each experiment, 20 mice were randomized in a 1:1 fashion to lovastatin or vehicle control, administered under isoflurane anesthesia (5 ml in a gas chamber), by oral gavage 3 hours prior to intra-tracheal bleomycin administration. During bleomycin administration, mice were anesthetized with subcutaneous injections of ketamine and tetracaine. The trachea was cannulated with a 22 gauge IV under direct visualization by tracheal cut-down procedure. Intra-tracheal bleomycin (0.025 U/20 g) was administered into the lungs. Tracheal incision was closed, and mice were recovered. Mice received daily administration of lovastatin (20 uM/kg) or vehicle (volume equivalent) by oral gavage following isoflurane anesthesia (5 ml in a gas chamber) until sacrifice at day 8 or day 14.

### Flow Cytometry Analysis

Mice were sacrificed at day 8; lungs were exsanguinated, removed, processed to single cell suspensions, and stimulated in vitro with propidium monoazide (PMA) and Ionomycin. 200,000 cells per well were plated on a 96 well plate and spun at 1000 rpm for 3 minutes. Liquid was removed, and cells were stained with 50 μL of FC Block/well diluted 1/100 in FACS buffer and allowed to incubate for 5 minutes in 4°C refrigerator. 50 μL of collagen/CD45 stain were added to each well (500 μL of FACS buffer was mixed with 2 μL of FITC labeled collagen stain and 1 mL of peridinin chlorophyll-a protein labeled (PerCP-labeled) CD45). Cell suspensions were refrigerated at 4°C for 10 minutes, and then 100 μL of FACS buffer was added to each well. Cells were spun at 1000 rpm for 3 minutes. Supernatant was removed, and 50 μL of phycoerythin (PE)-conjugated streptavidin was added to each well, along with 100 uL of FACS buffer, and incubated for 10 minutes at 4°C. 100 μL of FACS buffer was added to each well, and then the plate was spun for 3 minutes at 1000 rpm. Supernatant was removed and 100 μL of fixative was added to each well (3:1 diluent:concentrate). All flow cytometry was performed on a FacsCaliber, BD Biosciences (San Jose, CA, USA).

### Histology

Lungs were inflated to 20 cm of pressure with formalin, sectioned and stained for H&E and Masson-Trichrome stain. Samples were analyzed by microscope at 10× magnification.

### Statistics

For data to be reported in this paper a minimum of three independent experiments had to be performed on separate days with reproducible results. The chi-squared test was used to analyze survival data for the in vivo experiments. All other statistical analyses were conducted using the paired Student’s t-test. Statistically significant values were those where *p*<0.05.

## RESULTS

### LMW HA induces inflammatory protein expression in alveolar macrophages

**Figure 1:**
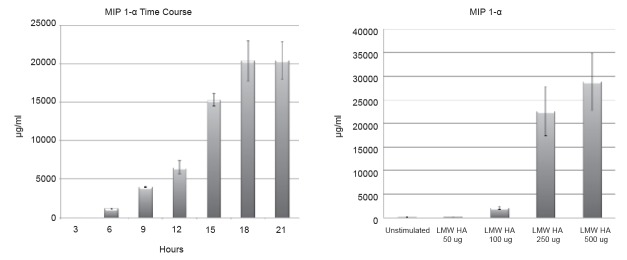
A, MH-S Alveolar Macrophages were stimulated with vehicle or LMW HA (250 μg/ml) with cell supernatants collected at various time points to establish a time course for peak MIP 1-α protein expression induced by LMW HA. Peak production occurs between 18-21 hours; B, MH-S Cells were stimulated with vehicle or varying doses of LMW HA with cell supernatants collected at 18 hours to establish the optimal dose of LMW HA to induce protein expression. Doses of 500 ug/ml induced more than 5% cell death by Trypan Blue Exclusion Assay in some experiments, so a dose of 250 ug/ml was considered the optimal dose to induce inflammatory expression.

MH-S Alveolar Macrophages were stimulated with vehicle or LMW HA (250 µg/ml) with cell supernatants collected every three hours to establish a time course for peak MIP 1-α protein expression induced by LMW HA. Peak production occurs between 18-21 hours, and the 18-hour mark served as a time point for collection of cell supernatants for protein analysis in all future experiments (Figure 1A). Next, MH-S Cells were stimulated with vehicle or varying doses of LMW HA with cell supernatants collected at 18 hours to establish the optimal dose of LMW HA to induce protein expression. Doses of 500 µg/ml induced more than 5% cell death by Trypan Blue Exclusion Assay in some experiments, so a dose of 250 µg/ml was considered the optimal dose to induce inflammatory expression (Figure 1B).

### Lovastatin exhibits immediate inhibition of LMW HA induced inflammatory cytokine protein expression

**Figure 2:**
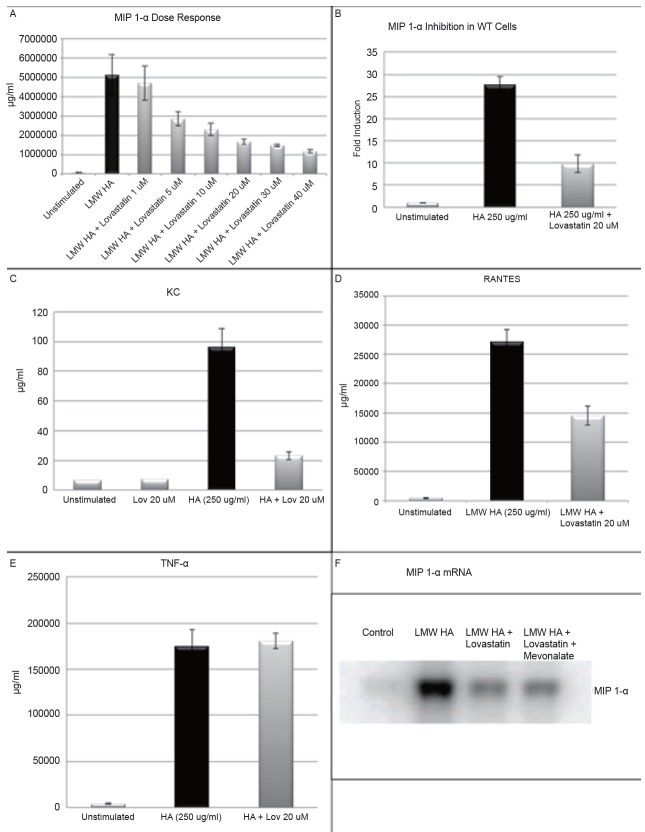
A, MIP 1-α protein by ELISA in MH-S alveolar macrophages treated with simultaneous administration of LMW HA (250 μg/ml) and varying doses of lovastatin (0, 1, 5, 10, 20, 30, and 40 μM). Lovastatin significantly inhibited MIP 1-α production with an IC50 noted at a dose of 5 μM (*p*=0.001); B, MIP 1-α protein by ELISA in Thioglycollate derived peritoneal macrophages treated with simultaneous administration of LMW HA (250 μg/ml) and lovastatin (20 μM). Lovastatin significantly inhibited MIP 1-α production; C-E, KC, RANTES and TNF-α protein by ELISA in MH-S alveolar macrophages treated with simultaneous administration of LMW HA (250 μg/ml) and lovastatin (20 μM). Lovastatin significantly inhibited KC (*p*=0.005), and RANTES (0.009) but not TNF-α expression; F, MH-S Cells were stimulated with vehicle, LMW HA (250 μg/ml) alone or with simultaneous administration of lovastatin (20 μM) or lovastatin (20 μM) and mevonalate (500 μM). MIP 1-α mRNA expression was measured by Northern Blot analysis. Lovastatin significantly inhibited LMW HA induction of MIP 1-α DNA, and the addition of mevonalate did not restore MIP 1-α mRNA expression.

Experiments were carried out in both wild type (WT) murine peritoneal derived macrophages and the MH-S cell line of murine alveolar macrophages. We initially determined the optimal inhibitory dose of lovastatin by stimulating MH-S alveolar macrophages with increasing doses of lovastatin ranging from 1 uM to 40 uM. Lovastatin was added to cell culture immediately prior to LMW HA (250 ug/ml) administration. MIP 1-α cytokine production was selected for primary analysis, because activation and cross-linking of LFA-1 has been shown to induce secretion of MIP 1-a leading to lymphocyte migration to areas of inflammation (27) . Protein supernatants were collected at 18 hours, and analyzed by ELISA for MIP 1-α cytokine production. We found that LMW HA markedly up-regulated MIP 1-α, and lovastatin inhibited this production in a dose dependent manner (Figure 2A). The IC_50_ appears to occur at a dose of 5 uM, but maximal inhibition occurred at a dose of 40 uM. Preliminary experiments demonstrated doses greater than 40 uM resulted in excessive cell death (>5%). To avoid a potential confounding effect a 20 uM dose (*p*=0.001) was determined to achieve a near maximal inhibition while avoiding the potential cytotoxic effects of higher doses.

We next evaluated the inhibitory effect of lovastatin in thioglycollate elicited wild type (WT) murine peritoneal macrophages. WT macrophages were treated with lovastatin (20 uM), immediately prior to LMW HA (250 ug/ml); protein supernatants were collected after 18 hours and analyzed for cytokine production. LMW HA resulted in a marked up-regulation of MIP 1-α, and treatment with lovastatin was able to attenuate this inflammatory response by nearly 15 fold (Figure 2B), confirming that our observation was not a cell line specific phenomenon. We then looked at additional inflammatory cytokines in MH-S alveolar macrophages treated with lovastatin (20 uM), immediately prior to LMW HA (250 ug/ml) stimulation. Statistically significant inhibition was seen with both RANTES (0.009) and KC (*p*=0.005) cytokine production; however, there was no inhibitory effect on TNF-α production (Figure 2C-E) Multiple papers have shown the ability of lovastatin to inhibit TNF-α expression, but at least one other paper has suggested that specific CD11a blockade may not inhibit TNF-α production in the lung (28, 29).

### Lovastatin inhibits inflammatory gene expression independent of HMG CoA reductase

MH-S murine alveolar macrophages were treated with vehicle control (lovastatin 20 µM and mevalonate 500 µM), LMW HA (250 µg/ml), LMW HA (250 µg/ml) with lovastatin (20 µM), or LMW HA (250 µg/ml) with lovastatin (20 µM) and mevalonate (500 µM) for a 6- hour time course. Cells were lysed and analyzed for MIP 1-α mRNA production by Northern blot (Figure 2F). In the presence of LMW HA, MIP 1-α mRNA production was markedly upregulated. Lovastatin inhibited MIP 1-α mRNA production, but mevalonate was unable to restore cellular production of MIP 1-α mRNA implicating an HMG-CoA independent mechanism of action for lovastatin.

### Pravastatin is unable to exhibit immediate inhibition of LMW HA induced cytokine protein expression

**Figure 3:**
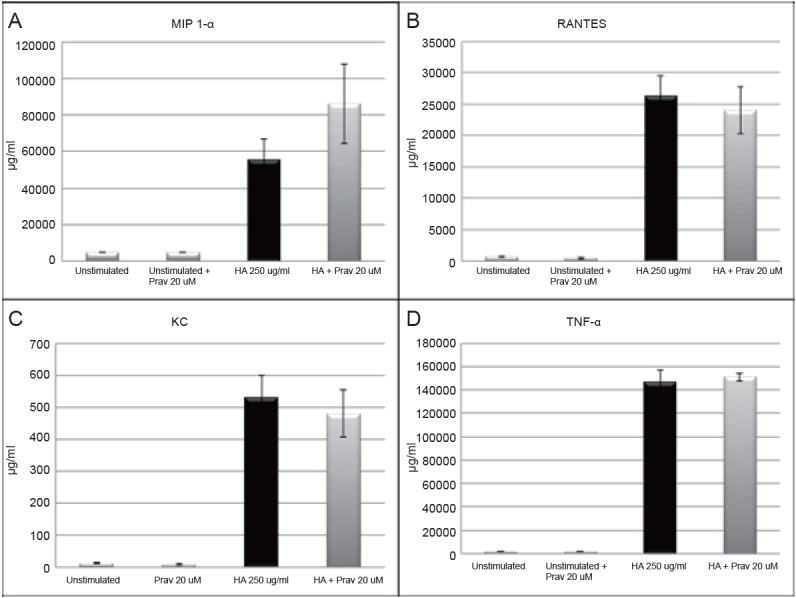
MH-S alveolar macrophages were simultaneously treated with pravastatin (20 μM) and LMW HA (250 μg/ml), and cytokine production was measured in cultured cell supernatents by ELISA. Simultaneous treatment with pravastatin was unable to attenuate the up-regulation of LMW HA induced cytokines (A. MIP 1-α, B. RANTES, C. KC, and D. TNF-α).

We next evaluated the inhibitory effect of pravastatin on LMW HA when given immediately prior to stimulation. MH-S murine alveolar macrophage cells were treated with pravastatin (20 uM) immediately prior to LMW HA (250 ug/ml) for an 18-hour incubation. Protein supernatants were collected and analyzed by ELISA for cytokine production. Pravastatin had no effect on LMW HA induced MIP-1α, RANTES, KC, or TNF-α (Figure 3A-D).

### Blocking LFA-1 inhibits LMW HA induced inflammatory cytokine protein expression

**Figure 4:**
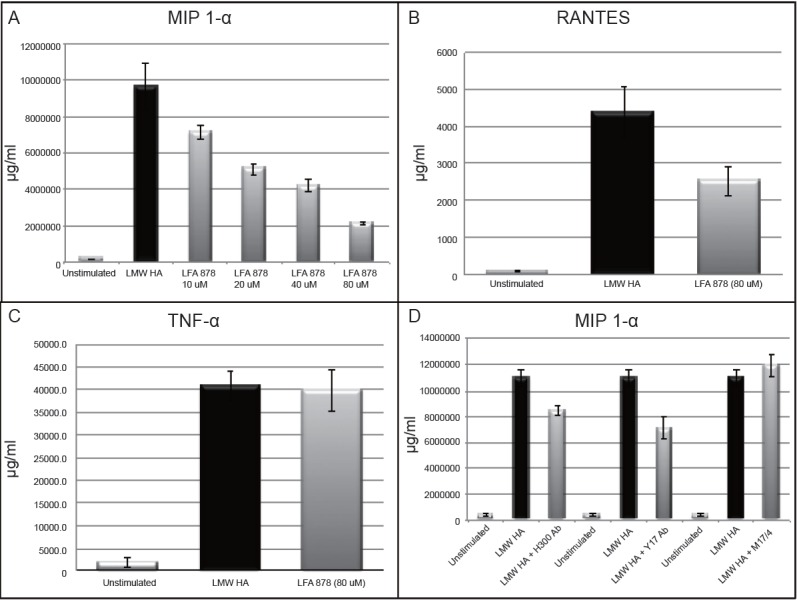
MH-S alveolar macrophage cells were simultaneously stimulated with the small molecule statin derivative LFA 878 and LMW HA (250 μg/ml) and cytokine production was measured. A, The small molecule statin derivative LFA 878 demonstrated a dose response inhibitory effect on LMW HA induced MIP 1-α expression with statistically significant inhibition noted at 20 μM (*p*=0.02) and maximal inhibition achieved at an 80 μM dose without evidence of cell death by Trypen blue assay (*p*=0.004); B) Simultaneous administration of LFA 878 (80 μM) also inhibited LMW HA induced RANTES production, although it did not reach statistical significance (*p*=0.08); C, There was no inhibitory effect of LFA 878 on LMW HA induced TNF-α production; D, Simultaneous administration of CD11a blocking antibodies (10 μg/ml) near the N-terminus of the I-domain inhibited LMW HA induction of MIP 1-α (Y17, *p*=0.022), as did an antibody directed towards talin protein that anchors LFA-1 to the cytoskeleton (H300, *p*=0.021). Whereas administration of M17/4 antibody specific for the carboxy-terminus of the I-domain of CD11a failed to inhibit LMW HA induction of MIP 1-α (*p*=0.428).

We utilized a novel low molecular weight statin derivative, LFA 878 (*Novartis, Basel-Switzerland*), to more specifically evaluate the role of LFA-1 in LMW HA mediated inflammatory signaling. LFA 878 was engineered to bind specifically to the I-domain of the CD11a arm of LFA-1. It has no reported effects on HMG CoA reductase activity, and has no other known epitopes (30). Alveolar macrophages were treated with LFA 878 at increasing doses prior to stimulation with LMW HA (250 ug/ml). Protein supernatants were collected at 18 hours and analyzed by ELISA for cytokine protein expression. LFA 878 inhibits MIP 1-α and RANTES expression but not TNF-a in a dose dependent manner (Figure 4A-C). Statistically significant inhibition occurred at a 40 uM dose (*p*=0.046). Cell death assessed by Trypan Blue exclusion assay was less than 5% at each dose.

We next utilized LFA-1 (CD11a) blocking antibodies to confirm the response seen with LFA 878. The I-domain of CD11a is reported to extend from amino acid 154-300 (31) . The Y17 antibody is specific for an N-terminal end near the 100 amino acid range, and the M17/4 antibody has an epitope near the C-terminal end of I-domain of the αL arm of the integrin (32, 33). The H300 antibody is reported to be specific for amino acids 800-1100 downstream from the I domain on CD11a, which is a mid-zone region where talin protein interacts and with LFA-1 to anchor it to the cytoskeleton (34) . Alveolar macrophages were pre-treated with these three anti-CD11a antibodies for 1 hour prior to stimulation with LMW HA, and the protein supernatants were collected at 18 hours and analyzed by ELISA for cytokine protein expression (Figure 4D). Our results suggest that different CD11a antibodies have different effects on LMW HA fragment induced MIP 1-α protein expression. Both Y17 and H300 antibodies were able to inhibit protein expression of MIP 1-α in response to LMW HA (*p*=0.022 and 0.021 respectively), while M17/4 showed no inhibitory property.

### Lovastatin attenuates bleomycin induced lung injury

**Figure 5:**
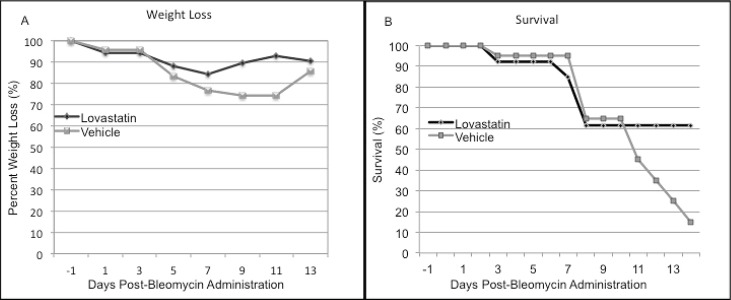
C57/BL6 mice were treated once daily with lovastatin 20 mg/kg by oral gavage one day prior to bleomycin lung injury (0.025 U/20g). Lovastatin inhibited (A) weight loss, and (B) mortality with an absolute mortality risk reduction of 45% at day 14 (n=20 in each group, χ^2^=10.6, *p*=0.014).

Bleomycin, a chemotherapeutic agent that has known pulmonary toxicity, is also known to increase total lung hyaluronan levels (35). Furthermore, transgenic animals that are unable to clear HA from the lungs due to lack of the HA receptor CD44 have increased damage and lung injury in response to bleomycin injury (12). We hypothesized that lovastatin, in part by inhibiting LMW HA induced inflammatory gene expression, would attenuate bleomycin-induced lung inflammation and fibrosis and improve overall mortality. WT mice were treated with lovastatin (20 mg/kg) or vehicle control (volume equivalent) by oral gavage beginning one day prior to intratracheal administration of bleomycin (0.025 U/20 g) and daily thereafter for 14 days. We monitored the mice for weight loss and survival. In the initial days following bleomycin administration weight loss was similar in both groups, but beginning on day 5 the lovastatin treated mice were noted to have less weight loss than our placebo group (Figure 5A). Animals treated with lovastatin had a clear survival advantage (ARR=45%) (Figure 5B). Thus, lovastatin attenuated mortality after bleomycin injury.

**Figure 6:**
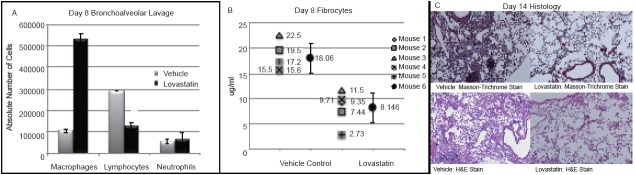
C57/BL6 mice were treated once daily with lovastatin 20 mg/kg by oral gavage one day prior to bleomycin lung injury (0.025 U/20g). A, Bronchoalveolar lavage at day 8, demonstrated significant differences between lovastatin and placebo treatment groups (n=3 in each group). Lovastatin treated mice had a markedly increased number of macrophages (*p*=0.002) and a statistically significant decrease in lymphocytes (*p*=0.01); B, The percentage of fibrocytes (CD45+/collagen+ cells) from whole lung lysates harvested at day 8 were also significantly decreased in lovastatin treated mice (*p*=0.001); C, Lovastatin treated mice demonstrated decreased histologic lung damage on hematoxylin and eosin stain with a decrease in collagen staining on Masson’s Trichrome Stain at day 14.

Lovastatin treatment also attenuated inflammation in bleomycin lung injury. Bronchoalveolar lavage (BAL) specimens on day 8 after bleomycin revealed statistically significant differences between absolute numbers of macrophages (*p*=0.002) and lymphocytes (*p*=0.01) between the treatment groups (Figure 6A). Lovastatin treated mice had increased numbers of macrophages but about half as many lymphocytes when compared to placebo.

In addition, we utilized flow cytometry for evaluation of whole lung lysates for pulmonary fibrocytes as a marker of active fibrosis (36) . Mice from three separate experiments in each group were sacrificed on day 8 and whole lung lysates were analyzed for fibrocytes by flow cytometry (CD45/Collagen double positive cells). The lovastatin treated group had significantly less pulmonary fibrocytes than their vehicle control (Figure 6B). Averaged across the group there was a statistically significant difference in lung fibrocytes at day 8 (*p*=0.001).

Lovastatin also attenuated histologic damage from bleomycin. We sacrificed mice 14 days after intratracheal instillation of bleomycin to evaluate for histopathologic evidence of lung injury. Lovastatin treatment markedly decreased inflammation, architectural damage and collagen deposition after bleomycin injury when compared to placebo treated animals (Figure 6C).

## DISCUSSION

In this study, we evaluated the effect of lovastatin, an HMG CoA reductase inhibitor, on inflammatory signaling and interstitial pulmonary inflammation leading to fibrosis induced by bleomycin lung injury. Our *in vivo* experiments confirmed that lovastatin treatment provides a survival advantage over placebo in bleomycin injured mice with decreased inflammation and decreased interstitial histologic damage (Figure 6). We also observed decreased circulating fibrocytes, suggesting that lovastatin inhibited recruitment of these cells to the injured lungs providing a potential anti-fibrotic therapeutic advantage. These data support a possible treatment effect of lovastatin in inflammatory interstitial lung diseases leading to pulmonary fibrosis.

The few existing animal studies looking at the treatment effect of statins in a model of bleomycin induced interstitial pulmonary fibrosis offer contradictory findings. The animal data on lipophilic statins would support a possible therapeutic role, as Ou and colleagues found survival benefits with simvastatin in bleomycin lung injury similar to our results with lovastatin. They demonstrated decreases in the histopathologic damage, as well as decreases in the pro-fibrotic cytokines transforming growth factor-beta1 (TGF-β1) and connective tissue growth factor (CTGF) (37). However, Xu *et al*. reported an increase in interstitial pulmonary fibrosis and pro-inflammatory cytokines in bleomycin injured mice pre-treated with pravastatin (38). In contrast, Kim *et al.* utilized supraphysiological doses of pravastatin at 30 mg/kg and 300 mg/kg, to reduce levels of TGF-β and CTGF resulting in decreased histopathological injury; however, there was no commentary on survival at these theoretically toxic doses (39).

Interestingly, observational clinical studies record contradictory findings as well. A recent study by Saad *et al*., examined records of 1.4 million patients from the Quebec health administrative databases, identified by their use of a respiratory inhaler, and found no association between statin use and the risk of interstitial lung disease in the 6000 plus cases of interstitial lung disease in the cohort (40). Unfortunately, Saad *et al*. did not make any further assessment to isolate hydrophilic statin use in their cohort, whereas Xu and colleagues, found that statin use in general, but hydrophilic statin use in particular, was associated with a statistically significant increase in radiologic interstitial abnormalities on thoracic CT imaging. (38) After adjustments for co-morbidities, only hydrophilic statin use continued to show a significant 4.5 fold increased risk of interstitial lung abnormalities. The etiology and nature of the interstitial abnormalities in Xu’s study are unknown, and the findings do not establish causality. Additionally, these studies were not designed as randomized controlled trials to test any therapeutic potential of statins in cases of pre-existing inflammatory interstitial lung disease, but do raise interesting questions regarding the potential differential effects of hydrophilic and lipophilic statins in interstitial lung disease. We set out to evaluate one potential mechanistic difference to determine if there may be a therapeutic target warranting further scientific study.

Since a dysregulated extracellular matrix is a pathological hallmark of interstitial pulmonary fibrosis, our *in vitro* experiments examined the potential of lipophilic statins to inhibit the cascade of inflammation induced by low molecular weight fragments of the extracellular matrix glycosaminoglycan hyaluronan (LMW HA). Prior studies have suggested the need for a period of pre-incubation to see the cellular anti-inflammatory effects of various statins, including lovastatin. We were able to show an immediate anti-inflammatory effect in response to LMW HA. This effect was not seen with pravastatin raising the possibility of a differential mechanism of action between lipophilic and hydrophilic statins. We evaluated whether repletion of the next immediate downstream product of HMG CoA reductase (mevalonate) would alter our results with lovastatin. Mevalonate did not restore normal cellular mRNA expression of MIP 1-α, in response to LMW HA. We concede that given the long incubation period necessary to evaluate protein expression, some of our results could conceivably be due to activity on HMG CoA reductase, and thus we cannot reliably attribute our findings with lovastatin to pure LFA-1 inhibition. However, we did achieve a similar inhibitory profile with the specific I domain LFA-1 inhibitor LFA 878, as well as an anti-CD11a antibody with activity near the N-terminal region of the I-domain of LFA-1. We also showed a similar inhibitory profile with a blocking antibody that could interfere with LFA-1 anchorage into the cytoskeleton suggesting an important role for LFA-1 in propagating the LMW HA inflammatory process. The findings with LFA 878 and the anti-CD11a antibodies, coupled with the fact that mevalonate could not restore mRNA gene expression, provide circumstantial evidence that LFA-1, and in particular the CD11a arm of LFA-1 is important for the full expression of MIP 1-α in response to LMW HA.

We have also hypothesized that lovastatin may have inhibited LFA-1/ICAM interaction, impairing fibrocyte migration, which resulted in the observed decrease in circulating fibrocytes homing to the lung in our animal model of pulmonary fibrosis. Our experimental design is unable to confirm or establish a definite mechanism for the observed survival advantage in lovastatin treated mice. It is unclear if direct inhibition of LMW HA induced inflammation contributed to the survival advantage, or if inhibition of LFA-1/ICAM interactions with impaired fibrocyte recruitment accounts for the observed mortality benefit, or if it is a combination of factors. Admittedly our results only provide plausible mechanistic hypotheses for the survival benefit seen with lovastatin. In that regard, direct comparisons of multiple lipophilic and hydrophilic statins in a bleomycin model of lung injury are needed to evaluate a differential inflammatory profile, as well as further exploration of the differential effect and role of fibrocyte recruitment to the lungs. Unfortunately, due to unforeseen circumstances we were unable to complete our own *in vivo* experiments with LFA 878, but further exploration of the therapeutic importance of specific I domain, LFA-1 inhibitors, such as LFA 878, in a model of bleomycin induced pulmonary fibrosis may reveal another important target for the management of this disease. These results at least lay some groundwork for future investigation of another therapeutic target for the management of inflammatory interstitial lung diseases leading to pulmonary fibrosis.

## CONCLUSIONS

Our data support an argument for the investigation of a therapeutic role for lipophilic statins in pulmonary fibrosis. We demonstrate a novel finding that direct inhibition of the I-domain of the CD11a arm of LFA-1 can attenuate extracellular matrix-macrophage induced inflammation *in vitro*, and *in vivo* lovastatin decreased fibrocyte recruitment and mitigated the histologic damage from bleomycin injury.

Although the therapeutic role of statins in inflammatory interstitial lung diseases leading to pulmonary fibrosis is unclear and at times contradictory, our experimental findings suggest a plausible mechanistic explanation for the observed clinical differences between lipophilic and hydrophilic statins in interstitial lung disease. Our findings also suggest a potentially beneficial treatment profile with lipophilic statins, possibly via LFA-1 inhibition, as well as adding to the speculation of the potential therapeutic role of direct LFA-1 I domain inhibitors in the management of pulmonary fibrosis.
